# Development of a scale to evaluate midwives’ beliefs about assessing alcohol use during pregnancy

**DOI:** 10.1186/s12884-015-0779-6

**Published:** 2015-12-30

**Authors:** Rochelle E. Watkins, Janet M. Payne, Tracy Reibel, Heather M. Jones, Amanda Wilkins, Raewyn Mutch, Carol Bower

**Affiliations:** Telethon Kids Institute, The University of Western Australia, Perth, Australia; Child and Adolescent Health Service, Department of Health Western Australia, Perth, Australia

**Keywords:** Midwives, Beliefs, Alcohol, Pregnancy, Prevention

## Abstract

**Background:**

Prenatal alcohol exposure is an important modifiable cause of adverse fetal outcomes during and following pregnancy. Midwives are key providers of antenatal care, and it is important to understand the factors which influence their ability to provide appropriate advice and support to women about alcohol use in pregnancy. The main aim of this study was to develop a psychometrically valid scale to evaluate midwives’ beliefs about assessing alcohol use during pregnancy.

**Method:**

A self-administered questionnaire was developed to evaluate midwives’ beliefs about assessing alcohol use during pregnancy, including beliefs about positive and negative consequences of asking about alcohol use, and beliefs about capacity to assess alcohol use. The questionnaire was sent to 245 midwives working for a state-wide country health service in Western Australia. Exploratory factor analysis was used to identify the latent constructs assessed by the 36 belief items and provide initial construct validation of the Asking About Alcohol (AAA) Scale.

**Results:**

Of the 166 (67.8 %) midwives who responded to the survey, 160 (96.4 %) completed one or more of the belief items and were included in this analysis. Factor analysis identified six subscales which assessed beliefs about discomfort, capacity, effectiveness, role, trust and knowledge. Midwives held the most positive beliefs about their capacity to ask and the effectiveness of asking about alcohol use, and the least positive beliefs about women’s knowledge about alcohol use and discomfort associated with asking about alcohol use in pregnancy. Midwives’ beliefs about their role and the effectiveness of asking were most strongly associated with the intention to ask all pregnant women about alcohol use during pregnancy (*r* = −0.59, *p* < 0.001 and *r* = −0.52, *p* < 0.001).

**Conclusions:**

Our analysis has identified key constructs underlying midwives’ beliefs about the assessment of alcohol use during pregnancy. The AAA Scale provides a basis for improved clarity and consistency in the conceptualisation and measurement of midwives’ beliefs which can be used to enhance our understanding of factors influencing midwives’ ability to deliver interventions to prevent alcohol use during pregnancy. The constructs identified in this exploratory analysis require confirmatory analysis to support their validity and generalizability.

**Electronic supplementary material:**

The online version of this article (doi:10.1186/s12884-015-0779-6) contains supplementary material, which is available to authorized users.

## Background

Fetal exposure to alcohol is associated with a range of negative outcomes, including placental dysfunction, stillbirth, premature birth, low birthweight, birth defects, growth restriction [1–4] and damage to the central nervous system, including the range of conditions collectively referred to as fetal alcohol spectrum disorder (FASD) [5, 6]. Despite this, studies in Australia and elsewhere have found that approximately half of women report drinking alcohol during pregnancy [7, 8], although recent evidence suggests this proportion is declining [9, 10].

Due to the absence of a known safe level of prenatal alcohol exposure, current Australian alcohol guidelines [11] recommend not drinking alcohol during pregnancy is the safest choice. This differs from the previous guidelines which recommended consumption of less than 7 standard drinks per week, and no more than 2 standard drinks on any one day [12]. Recommendations alone are unlikely to be sufficient to facilitate changes in drinking patterns among pregnant women, and a range of interventions [13–15], and practice guidelines for health professionals [16, 17] have been developed to promote awareness of the current guidelines and support abstinence from alcohol use during pregnancy.

Consistent with international guidelines for the identification and management of substance use in pregnancy [18], Australian national clinical practice guidelines for antenatal care recommend the assessment of prenatal alcohol use for all women and the use of brief intervention to prevent prenatal alcohol use [17]. Despite considerable support for the implementation of screening and brief intervention for alcohol consumption among pregnant women in Australia [16, 17], there has been little evaluation of the factors which influence the implementation of these interventions by primary providers of antenatal care.

Our understanding of the factors which influence health professionals’ behaviour is incomplete [19], and there is a recognised gap between evidence-based recommendations and practice [20]. Midwives have been identified as important providers of alcohol education [21] due to their key role in assessing the risks associated with alcohol use during pregnancy and providing appropriate interventions to mitigate risks to maternal and fetal health. However, few studies have examined the factors which influence midwives’ ability to deliver interventions to prevent alcohol use during pregnancy.

Research in the United Kingdom has found midwives’ attitude towards advising pregnant women about alcohol use, including whether this was considered helpful, important and satisfying, was the strongest predictor of intention to advise women about the adverse effects of alcohol use [21]. Midwives were conscious of the tension between the need to address alcohol use while maintaining their unique relationship with women in their care, and described the need for high level communication skills and clinical judgement to achieve this balance [21]. A small qualitative study of midwives and pregnant women in Australia also found that informing pregnant women about the risks of alcohol use during pregnancy and providing risk reduction advice or referral was considered by both midwives and pregnant women to be an important part of the midwives’ role [22]. As a result, these authors suggest that reported fears of damaging the patient-provider relationship through asking about alcohol use may be unfounded. However, the study also found that midwives rarely discussed specific risks associated with alcohol use with pregnant women to avoid causing distress, particularly when alcohol had been consumed prior to the confirmation of pregnancy [22].

A perceived lack of evidence in the area has been identified as a barrier to discussing the risks associated with prenatal alcohol use [22]. A survey of midwives in Sweden found midwives believed that improved knowledge about the use of conversational techniques, screening instruments and improved guidelines for how to address alcohol with expectant parents and risky drinkers could facilitate increased intervention to prevent alcohol exposure during pregnancy [23]. A subsequent Swedish study indicated that a national educational intervention was successful in enhancing midwives’ knowledge and implementation of screening and provision of advice about the health risks associated with alcohol use in pregnancy [24].

An improved understanding of midwives’ beliefs about assessing alcohol use during pregnancy can be used to identify their support needs and develop targeted interventions to facilitate best practice among these key service providers. However, the specific beliefs assessed have varied between studies, and there is a need to identify the salient beliefs among midwives, improve the clarity of the constructs measured, and facilitate the development of specific targets for intervention to promote attitude and behaviour change. The aim of this study was to develop a psychometrically valid scale to evaluate midwives’ beliefs about the assessment of alcohol use during pregnancy, and to examine the association between these beliefs, individual characteristics, and established predictors of behaviour including attitudes to asking about alcohol use and intention to ask about alcohol use during pregnancy.

## Methods

This paper reports on the initial development of the Asking About Alcohol (AAA) Scale to assess beliefs about the positive and negative consequences of asking about alcohol use, and the perceived capability to ask about alcohol use during pregnancy. The following steps were used to develop and evaluate the AAA Scale: specification of the theoretical framework for the study, item development and expert review, administration of the scale items among midwives working in a state-wide country health service, exploratory factor analysis to identify the underlying factor structure of the scale, and assessment of subscale internal consistency and construct validity.

## Theoretical framework

To support its content validity, scale construction was guided by relevant theory and concepts identified in previous studies on the prevention of alcohol use in pregnancy. Despite calls for the use of theory to guide the development of interventions to improve the implementation of evidence-based practice, the atheoretical nature of many interventions has been understood as a consequence of the number and overlapping nature of theories and their constructs [25]. Although there is as yet no clearly identified theoretical model of choice to understand midwives’ practices to prevent alcohol use during pregnancy, the Theory of Planned Behaviour [26] was selected as the primary theoretical basis for scale development based on the growing body of evidence supporting the utility of this theory to explain health professionals’ behaviour [19]; the previous application of this theory to explain midwives’ assessment of alcohol use during pregnancy [21]; and consistency evident between this approach and others including the recently revised Theoretical Domains Framework, which was developed to simplify and combine knowledge derived from a number of behaviour change theories [27].

A systematic review of 78 studies which used social cognitive theories to predict health professionals’ intentions and behaviours found the Theory of Planned Behaviour was an appropriate and frequently used theory to predict behaviour [19]. Health professionals’ beliefs about capabilities were found to most consistently predict their behaviours, and beliefs about capabilities and consequences were found to most consistently predict their behavioural intentions [19]. These key constructs are also recognised as important determinants of behaviour in the recently revised Theoretical Domains Framework [27], which is receiving increasing attention as a means of understanding factors which affect implementation of interventions among health professionals, and has been found to provide a comprehensive understanding of the barriers to and facilitators of the uptake of clinical interventions [28]. The Theoretical Domains Framework constructs most relevant to and consistent with specific beliefs underlying perceptions of attitudes and perceived behavioural control are beliefs about consequences and beliefs about skills. Further theory-based research has been recommended to develop tools to accurately assess barriers and facilitators to the implementation of interventions in the clinical setting [28].

Single-item indicators of beliefs about capabilities and consequences have been examined in a large study of health professionals in Western Australia: ‘It is easy to ask pregnant clients about how much and how often they drink alcohol’ (capabilities); ‘Discussing alcohol use during pregnancy will frighten or anger pregnant women’ (negative consequences); and ‘Advising women who are pregnant to avoid binge drinking may reduce fetal alcohol syndrome’ (positive consequences) [29]. Similar concepts have been identified as important determinants of midwives’ practice in other contexts [21, 22, 30]. However, no consistent approach to the assessment of health professionals’ beliefs about assessing alcohol use in pregnancy has been established, leading to a lack of clarity in the identification of the latent constructs underlying these beliefs, and limiting the identification of implications for service development.

## Item development and expert review

Based on a conceptual analysis of relevant theoretical constructs and review of research findings, items were developed to assess salient beliefs about positive and negative consequences of asking about alcohol, and capability to ask about alcohol use during pregnancy. Following development of the initial item pool, expert content validation was sought. The proposed scale items were reviewed for comprehensiveness, clarity and face validity by: 7 members of the study steering group who had relevant content expertise in alcohol and pregnancy research and practice and included a consumer who provided a community perspective on the study; 8 members of a midwifery reference group, comprised of 6 midwifery leaders and 2 clinical midwives; and by 6 other specialist reviewers, including reviewers with expertise in the delivery of brief intervention for alcohol use during pregnancy and health service development. Based on feedback from the expert reviewers, one item was added to the scale, and the final pool of 36 items was considered to capture relevant and salient beliefs.

## Questionnaire design

A self-administered questionnaire (Additional file 1) was developed which incorporated the 36 belief items, and items which assessed sociodemographic characteristics and variables used to examine the concurrent validity of the beliefs assessed. All belief items were assessed on a 7-point Likert scale with anchors ‘strongly agree’ and ‘strongly disagree’. For analysis, negatively worded statements were reverse-scored so that a higher score represents more positive beliefs (disagreement with negative consequences, agreement with positive consequences, and agreement with capacity to assess alcohol use). The hard copy questionnaire was pretested with 5 members of the Australian College of Midwives WA Branch, and the online questionnaire was pilot tested with a further 20 participants from the Australian College of Midwives WA Branch and 5 child health researchers.

## Questionnaire administration

We surveyed midwives employed by a state-wide country health service in Western Australia (WA). In June 2013 all midwifery managers were emailed a description of the study, which included a study information sheet about the research including information on risks and benefits of participation, a link to an online version of the study questionnaire developed using Qualtrics [31], and a pdf copy of the questionnaire suitable for printing. Managers were asked to forward the email all to midwives working in their line of management involved in delivering clinical midwifery services inviting them to participate in the research. After two weeks midwifery managers were asked to forward a second email invitation to their staff to improve participation. Hard copies of the questionnaire were also subsequently distributed to managers to improve response. Managers were asked to report the number of eligible staff who were sent or given the questionnaire.

## Data management and analysis

Hardcopy questionnaires received were entered using the online questionnaire form, and online questionnaire data were downloaded in SPSS format. Exploratory factor analysis was used to identify the latent constructs assessed by the 36 belief statements. Factor extraction was based on the findings of parallel analysis [32] alongside examination of the screen plot and interpretability of the emergent factors. Factor extraction was performed using Principal Axis Factoring, and was compared with maximum likelihood and principal components solutions to identify areas of divergence. Oblique rotation was selected based on the expected association between factors, and was performed using Promax. Correlations between factors are described. Scale refinement retained items where the primary factor loading was 0.4 or greater, and items were removed where cross-loadings were not less than the primary loading minus 1.5 [33, 34]. Factors with two items were only retained where the items were highly correlated (*r* > 0.7), were only weakly correlated with other variables, and had a clear interpretation [34].

Chronbach’s alpha was used to assess the internal consistency of the subscales, and subscale scores were calculated as an average score for each subscale. Concurrent validity of the subscales was examined through assessment of the association between subscale scores and individual characteristics (age in years, number of years worked as a midwife), intention to ask all pregnant women about whether they have consumed alcohol during pregnancy, attitudes to asking about alcohol use (usefulness, pleasantness), social norms (pregnant women expect me to ask, my manager expects me to ask), perceived behavioural control (I am confident to ask), and beliefs about alcohol use (pregnant women should completely abstain, and infrequent drinking is not harmful). Differences in subscale scores were also assessed according to completion of brief intervention training on alcohol consumption within the last 2 years, reported use of the health service standard alcohol screening tool, and whether midwives ever see women in the first trimester of pregnancy. Spearman rank correlation coefficients and independent t-tests were used to examine the association between subscale scores and the other variables assessed. Analysis was performed using IBM SPSS Statistics for Windows, Version 22.0 (IBM Corp., 2013, Armonk, NY). This study was approved by the Western Australian Country Health Service Board Research Ethics Committee. Receipt of a completed questionnaire was approved as evidence of informed consent to participate in the research by the ethics committee.

## Results

Of the 245 midwives invited to participate in the study, 166 (67.8 %) returned the questionnaire, and 160 (65.3 %) completed one or more of the 36 belief scale items. The characteristics of those who completed one or more of the 36 belief scale items are summarised in Table 1. Participants ranged in age from 22 to 67 years (mean 46.2, sd 11.2 years) and reported working as a midwife for between less than 1 year to 44 years (mean 16.9 sd 11.5 years). Most participants (61.5 %) reported graduating from midwifery education prior to the year 2000. No sociodemographic data were available for non-participants.

**Table 1:** Summary of participant characteristics

Characteristic	*n* (%)^a^
Sex (*n* = 159)	
Female	157 (98.7)
Male	2 (1.3)
Main region of work (*n* = 158)	
South	76 (48.1)
Central	48 (30.4)
North	34 (21.5)
Age category (*n* = 154)	
20–29 years	19 (12.3)
30–39 years	23 (14.9)
40–49 years	37 (24.0)
50–59 years	61 (39.6)
60–69 years	14 (9.1)
Years worked as a midwife (*n* = 159)	
Less than 10	52 (32.7)
10 or more	107 (67.3)
Years worked as a midwife in the WACHS (*n* = 157)	
Less than 10	109 (69.4)
10 or more	48 (30.6)
Model of care (*n* = 145)	
Hospital-based team	78 (53.8)
General practitioner-based (shared care or general practitioner-led care)	51 (35.2)
Other (team or community midwifery, caseload midwifery, antenatal midwives clinic)	16 (11.0)
Completed specific training on BI at orientation (*n* = 155)	
Yes	40 (25.8)
No	115 (74.2)
Completed specific training on BI within the last 2 years (*n* = 154)	
Yes	54 (35.1)
No	100 (64.9)
Aware of policy on assessment of alcohol consumption during pregnancy (*n* = 151)	
Yes	99 (65.6)
No	52 (34.4)

^a^Proportion of valid responses

*General practitioner* general medical practitioner

*WACHS* Western Australian Country Health Service

*BI* brief intervention for alcohol use during pregnancy

## Factor structure of the beliefs items

Parallel analysis, examination of the scree plot, and examination of the interpretability of a range of factor solutions all indicated that extraction of a six factor solution was most appropriate. The initial rotated factor solution is presented in Table 2. Following the iterative removal of items with weak primary loadings or items which loaded on more than one factor, the final rotated factor solution was generated (Table 3). The 6 factors extracted explained 59.8 % percent of the total variance in scores, and the internal consistency of the 30-item scale was 0.87.

**Table 2:** Exploratory factor analysis of the belief items - initial rotated factor solution (*n* = 151)

	Factor					
Item (*n* = 36)	Discomfort	Capacity	Effectiveness	Role	Trust	Knowledge
Asking pregnant women about whether they have consumed alcohol during pregnancy can make them feel uncomfortable	**.780**					
Will cause anxiety and guilt among women who have consumed alcohol during pregnancy	**.769**	-.122		-.106		
Pregnant women don’t like to be asked about whether they have consumed alcohol during pregnancy	**.721**	-.104				
Will lead to some women feeling judged	**.710**	.183	.199	-.163	-.144	
Will distress or anger pregnant women	**.690**		.163	-.101		
Could make women fear losing custody of their child	**.627**				-.142	
Asking pregnant women whether they have consumed alcohol during pregnancy could appear judgemental	**.561**				.325	
I don’t like asking pregnant women to describe their behaviours which may have harmed their child	**.547**	.224				
Will threaten my relationship with pregnant women	**.521**	.234				.160
I have doubts about whether I am doing the right thing when pregnant women who have only consumed small amounts of alcohol experience guilt after discussing their alcohol consumption	**.470**			.241	.151	
Will uncover complex problems that are difficult for us to address as midwives	**.428**		-.229			
Offers little real benefit for women who are not in high risk groups	.298	-.130	.225	.149		
Women who have consumed alcohol during pregnancy will consider terminating their pregnancy if asked about their alcohol consumption	.206	.148		.113		.170
When I discuss the effects on the fetus and child of alcohol consumption during pregnancy I know what to say (R)		**.935**		-.136		
I have sufficient referral resources to adequately address alcohol use problems once identified (R)	-.106	**.723**	.167	-.199		
I have a good understanding of the evidence for the recommendation of no alcohol in pregnancy (R)		**.599**		.284		-.144
I always know what to say when I ask pregnant women about whether they have consumed alcohol during pregnancy (R)		**.484**	-.154	.203	.270	
I know exactly what I need to do to ask pregnant women about whether they have consumed alcohol during pregnancy (R)		**.430**	.216	.125	.159	
Improves women’s awareness of the importance of not consuming alcohol during pregnancy (R)	-.114		**.698**	-.180	.109	
Will identify women who need support to stop consuming alcohol during pregnancy (R)			**.688**			
Is necessary for me to provide appropriate advice and support (R)			**.635**			
Will enable changes in behaviour and improved health outcomes for the mother and child (R)			**.606**	.184		
Brief intervention (assessment, feedback, counselling and referral to a specialist if necessary) to decrease alcohol exposure in pregnancy is effective (R)		.262	**.492**			
Will make it easier to diagnose or exclude alcohol related problems in the child (R)		.127	.318	.191	-.167	
Documentation of any amount of alcohol exposure in pregnancy is important (R)	-.111			**.987**		
Identifying all women who drink any amount of alcohol during pregnancy is good practice (R)				**.731**		
Identifying all women who drink alcohol during pregnancy can help to improve health outcomes (R)		.177	.165	**.510**	-.224	.124
I don’t have sufficient time to ask every pregnant woman about whether they have consumed alcohol during pregnancy	.115	-.267		**.490**	.206	
I am uncomfortable asking pregnant women about their alcohol consumption before establishing a relationship of trust with them		-.151	.207	-.101	**.800**	
I am uncomfortable asking pregnant women about their alcohol consumption when it is the first time I have met them					**.600**	
I am uncomfortable asking pregnant women about their alcohol consumption as I may have a responsibility to notify the Department of		.243	-.126		**.551**	
Child Protection						
When I feel short of time I am less likely to ask pregnant women about whether they have consumed alcohol during pregnancy					.386	-.201
It is difficult to ask pregnant women about alcohol consumption during pregnancy without good knowledge of the effects of alcohol consumption during pregnancy on the fetus and child				-.242	.379	
I have the required skills to ask every pregnant woman about whether they have consumed alcohol during pregnancy (R)		.345		.126	.353	
Most pregnant women already have good knowledge about alcohol consumption in pregnancy	-.121					**.920**
Most pregnant women know not to drink alcohol during pregnancy						**.766**

(R) Scoring reversed for positively worded items

Loadings ≥ 0.4 are presented in bold

Loadings < 0.1 not shown

**Table 3:** Exploratory factor analysis of the belief items - final rotated factor solution (*n* = 151)

	Factor					
Item (*n* = 30)	Discomfort	Capacity	Effectiveness	Role	Trust	Knowledge
1. Will cause anxiety and guilt among women who have consumed alcohol during pregnancy	**.802**	-.106				
2. Asking pregnant women about whether they have consumed alcohol during pregnancy can make them feel uncomfortable	**.780**					
3. Pregnant women don’t like to be asked about whether they have consumed alcohol during pregnancy	**.732**	-.144				
4. Will distress or anger pregnant women	**.721**		.177			
5. Will lead to some women feeling judged	**.710**	.176	.190	-.140	-.147	
6. Could make women fear losing custody of their child	**.633**				-.163	
7. Asking pregnant women whether they have consumed alcohol during pregnancy could appear judgemental	**.592**				.268	
8. Will threaten my relationship with pregnant women	**.527**	.205				.147
9. I don’t like asking pregnant women to describe their behaviours which may have harmed their child	**.506**	247	-.114		.149	.100
10. I have doubts about whether I am doing the right thing when pregnant women who have only consumed small amounts of alcohol experience guilt after discussing their alcohol consumption	**.464**			.236	.170	
11. Will uncover complex problems that are difficult for us to address as midwives	**.423**		-.241			
12. When I discuss the effects on the fetus and child of alcohol consumption during pregnancy I know what to say (R)		**.935**		-.105		
13. I have sufficient referral resources to adequately address alcohol use problems once identified (R)	-.102	**.704**	.157	-.158		
14. I have a good understanding of the evidence for the recommendation of no alcohol in pregnancy (R)		**.593**		.291		-.126
15. I always know what to say when I ask pregnant women about whether they have consumed alcohol during pregnancy (R)		**.444**	-.138	.221	.254	
16. I know exactly what I need to do to ask pregnant women about whether they have consumed alcohol during pregnancy (R)		**.403**	.219	.152	.148	-.100
17. Improves women’s awareness of the importance of not consuming alcohol during pregnancy (R)			**.671**	-.151	.103	
18. Will identify women who need support to stop consuming alcohol during pregnancy (R)			**.660**			
19. Is necessary for me to provide appropriate advice and support (R)			**.630**			
20. Will enable changes in behaviour and improved health outcomes for the mother and child (R)			**.598**	.233		
21. Brief intervention (assessment, feedback, counselling and referral to a specialist if necessary) to decrease alcohol exposure in pregnancy is effective (R)		.278	**.480**			
22. Documentation of any amount of alcohol exposure in pregnancy is important (R)	-.107			**.976**		
23. Identifying all women who drink any amount of alcohol during pregnancy is good practice (R)				**.751**		
24. Identifying all women who drink alcohol during pregnancy can help to improve health outcomes (R)		.178	.159	**.503**	-.174	.128
25. I don’t have sufficient time to ask every pregnant woman about whether they have consumed alcohol during pregnancy	.131	-.250		**.447**	.198	
26. I am uncomfortable asking pregnant women about their alcohol consumption before establishing a relationship of trust with them		-.141	.207	-.122	**.811**	
27. I am uncomfortable asking pregnant women about their alcohol consumption when it is the first time I have met them	-.131				**.726**	
28. I am uncomfortable asking pregnant women about their alcohol consumption as I may have a responsibility to notify the Department of Child Protection		.229	-.124		**.521**	
29. Most pregnant women already have good knowledge about alcohol consumption in pregnancy	-.124					**.869**
30. Most pregnant women know not to drink alcohol during pregnancy						**.822**

(R) Scoring reversed for positively worded items

Loadings ≥ 0.4 are presented in bold

Loadings < 0.1 not shown

The first factor, beliefs about discomfort, accounted for the highest proportion of variance in scale scores (26.0 %). All 11 items with primary loadings on this factor described beliefs about the negative emotional consequences of asking about alcohol use, including negative emotional consequences for both pregnant women and midwives. Items loading most strongly on this factor described negative emotional consequences for pregnant women, including anxiety, discomfort and distress. The second factor, beliefs about capacity, accounted for 11.5 % of the variance in scale scores. All 5 items with primary loadings on this factor described beliefs about the capacity to ask about alcohol use during pregnancy, including the two highest loading items which assessed beliefs about knowing what to discuss and having sufficient resources for referral.

The third factor, beliefs about effectiveness, accounted for 7.2 % of the variance in scale scores. Items with primary loadings on this factor described beliefs about positive consequences of asking about alcohol use during pregnancy, including improving women’s awareness, identifying women who require support, and enabling change. The fourth factor, beliefs about role, accounted for 6.2 % of the variance in scale scores. Items with primary loadings on this factor described beliefs about their role in asking about alcohol use during pregnancy, including the need to identify any amount of use, and that identifying all women who consume any amount of alcohol is consistent with good practice.

The fifth factor, beliefs about trust, accounted for 4.7 % of the variance in scale scores. Items with primary loadings on this factor described beliefs relevant to the importance of establishing a relationship of trust between women and the midwife. The sixth factor, beliefs about knowledge, accounted for 4.2 % of the variance in scale scores. Both items with primary loadings on this factor described beliefs about women’s existing level of knowledge about the need to abstain from alcohol use during pregnancy.

The internal consistency of all factors exceeded 0.70, and correlations between factors are shown in Table 4. The strongest correlations observed between factors are consistent with the common constructs used to guide item-development. The strongest correlations were observed between factors 1 (discomfort) and 5 (trust) (*r* = 0.60), with these constructs assessing different aspects of beliefs about negative consequences associated with asking about alcohol use; and factors 2 (capacity) and 4 (role) (*r* = 0.53), with these constructs both assessing different aspects of beliefs relevant to the role and capability of midwives to ask about alcohol use.

**Table 4:** Factor correlation matrix for the Asking About Alcohol (AAA) Scale

Subscale	Discomfort	Capacity	Effectiveness	Role	Trust	Knowledge
1. Discomfort	(0.87)					
2. Capacity	.29	(0.84)				
3. Effectiveness	.29	.35	(0.77)			
4. Role	.34	.53	.39	(0.74)		
5. Trust	.60	.37	.28	.46	(0.70)	
6. Knowledge	-.08	-.33	-.13	-.12	-.08	(0.84)

Chronbach’s alpha shown on diagonal

Mean scores for each of the subscales are presented in Table 5, and indicate that midwives surveyed held the most positive beliefs about their role and effectiveness, and held the least positive beliefs about women’s knowledge and discomfort, with mean subscale scores around or below the scale median of 4 (‘neither agree nor disagree’). In contrast to the mode of 7 (‘strongly agree’) for midwives’ scores on factors 3, 4 and 5, scores on factor 6 had a mode of 3, scores on factor 1 had a mode of 4, and scores on factor 2 had a mode of 5.5.

**Table 5:** Descriptive statistics for belief subscales of the Asking About Alcohol (AAA) Scale

Subscale	Items	Minimum	Maximum	Median	Mean (sd)	95 % CI mean	Skewness	Kurtosis
1. Discomfort	11	1	7	4.5	4.4 (1.2)	4.3–4.6	−0.11	−0.41
2. Capacity	5	1	7	5.6	5.4 (1.2)	5.2–5.5	−0.95	1.06
3. Effectiveness	5	3	7	6.2	6.0 (0.9)	5.9–6.2	−0.87	0.48
4. Role	4	2	7	6.5	6.2 (0.9)	6.1–6.4	−1.79	4.66
5. Trust	3	1	7	5.3	5.2 (1.5)	4.9–5.4	−0.51	−0.64
6. Knowledge	2	1	7	3.0	3.5 (1.7)	3.3–3.8	0.43	−0.74

*CI* confidence interval

## Associations between subscale scores and other variables assessed

Associations between subscale scores and other variables assessed are summarised in Table 6. We found a weak positive association between age and beliefs about capacity to ask about alcohol use. The intention to ask all pregnant women about alcohol use was moderately positively correlated with 5 of the 6 subscale scores, with the strongest associations observed with beliefs about role and effectiveness. The perceived usefulness of asking about alcohol was associated with 4 of the 6 belief subscales, and most strongly associated with beliefs about effectiveness. The perceived pleasantness (for midwives) of asking about alcohol was associated with 5 of the 6 belief subscales, and most strongly associated with beliefs about discomfort. The belief that pregnant women expect midwives to ask about alcohol use were associated with all 6 beliefs subscales, and most strongly associated with beliefs about capacity and beliefs about women’s knowledge. The belief that their manager expects them to ask about alcohol use was associated with 4 of the 6 beliefs subscales, and most strongly associated with beliefs about role. The belief that women should completely abstain from alcohol use in pregnancy was most strongly associated with beliefs about role, effectiveness and capacity. The belief that infrequent alcohol use in pregnancy is not harmful was most strongly associated with beliefs about role, discomfort and effectiveness.

**Table 6:** Correlations between subscale scores for the Asking About Alcohol (AAA) Scale and age, experience, and theoretically related constructs

	Subscale					
Variable	Discomfort	Capacity	Effectiveness	Role	Trust	Knowledge
Age (years)	0.00	0.17*	0.11	0.07	−0.01	0.06
Years worked as a midwife	0.07	0.08	0.06	0.11	−0.05	0.14
Intention to ask about alcohol use	0.43***	0.40***	0.52***	0.59***	0.36***	−0.05
Attitudes to asking about alcohol use:						
Useful	0.22**	0.16	0.39***	0.33***	0.26**	−0.01
Pleasant	0.42***	0.18*	0.30***	0.29***	0.21*	−0.02
Social norms:						
Pregnant women expect me to ask	0.19*	0.44***	0.32***	0.36***	0.22**	−0.39***
My manager expects me to ask	0.15	0.24**	0.31***	0.52***	0.22**	−0.11
Perceived behavioural control:						
I am confident to ask	0.29**	0.45**	0.35**	0.47**	0.46**	−0.13
Beliefs about alcohol consumption:						
Pregnant women should completely abstain	0.18*	0.30***	0.31***	0.49***	0.24**	−0.03
Infrequent drinking is not harmful	−0.36***	−0.18*	−0.34***	−0.37***	−0.29***	−0.12

Spearman rank correlation coefficients reported

Due to missing data, numbers vary between 149 and 159

*p < 0.05; **p < 0.01; ***p < 0.001

Midwives who reported completion of brief intervention training for alcohol use in the previous 2 years reported more positive beliefs about capacity (mean = 5.7) than midwives who had not completed training (mean = 5.2) (t_(151)_ = 2.9, *p* = 0.005, mean difference = 0.55 (95 % confidence interval (CI) 0.17–0.93), Cohen’s d = 0.47). Midwives who reported using the standard alcohol screening tool recommended in the health service studied, reported more positive beliefs about capacity (mean = 5.7) than midwives who did not report using the screening tool (mean = 5.2) (t_(136)_ = 2.8, *p* = 0.007, mean difference = 0.52 (95 % CI 0.15–0.89), Cohen’s d = 0.48); and more positive beliefs about effectiveness (mean = 6.3) than midwives who did not report using the screening tool (mean = 5.9) (t_(137)_ = 2.8, *p* = 0.006, mean difference = 0.41 (95 % CI 0.12–0.70), Cohen’s d = 0.48). Midwives who reported ever seeing pregnant women during their first trimester of pregnancy reported more positive beliefs about capacity (mean = 5.6) than midwives who did not see pregnant women during their first trimester of pregnancy (mean = 4.9) (t_(149)_ = 3.4, *p* = 0.001, mean difference = 0.68 (95 % CI 0.28–1.07), Cohen’s d = 0.56).

## Discussion

We report the development of a scale to evaluate midwives’ beliefs about assessing alcohol use during pregnancy. Our exploratory analysis of the factor structure of the AAA Scale found the scale assesses constructs which reflect both positive and negative consequences of asking about alcohol use in pregnancy through the identification of the factors labelled discomfort, trust, effectiveness and knowledge; and capability to ask about alcohol use in pregnancy through the identification of the factors labelled capacity and role. The belief constructs identified through exploratory factor analysis are consistent with constructs identified as salient in previous studies of midwives beliefs about screening and brief intervention for alcohol use during pregnancy [21–23, 30]. Beliefs about capacity and consequences have also been identified as important predictors of behaviour in the Theory of Planned Behaviour and the Theoretical Domains Framework [19, 27].

Midwives held positive beliefs about their role in assessing alcohol use during pregnancy, their capacity to ask, and the effectiveness of asking to improve awareness of the importance of abstaining from alcohol use during pregnancy and enable behaviour change to improve the health of the woman and fetus. However, midwives held less positive beliefs about women’s knowledge about alcohol use in pregnancy. Many midwives identified discomfort associated with asking about alcohol use during pregnancy that was related to potential negative emotional consequences both for pregnant women and for themselves. Beliefs about discomfort accounted for over a quarter of the total variance in scale scores. These findings indicate the need to address midwives’ concern about the potential to cause discomfort when asking about alcohol use in the design and implementation of policies on screening and brief intervention for alcohol use in pregnancy. More widespread use of screening and brief intervention to improve pregnant women’s knowledge about alcohol use may address midwives’ concerns that women do not have a good knowledge of alcohol use during pregnancy.

The moderate associations found between beliefs assessed by the AAA Scale and indicators of constructs central to behavioural theory including attitudes, social norms, perceived behavioural control and behavioural intentions [26, 27] suggest that the beliefs assessed by the AAA Scale identify constructs that are relevant to understanding midwives’ behaviour in relation to asking about alcohol use. An understanding of these beliefs will provide information relevant to the design and evaluation of interventions to promote practice consistent with clinical practice guidelines. Midwives’ intentions to ask about alcohol use were moderately correlated with all belief subscales apart from knowledge, and attitudes to asking were most strongly related to beliefs about effectiveness and discomfort. These findings support the use of behavioural theory to understand midwives’ practice and indicate the need for further research to investigate the application of theoretically-based interventions to improve midwives’ capacity to prevent prenatal alcohol exposure.

Like the Theoretical Domains Framework constructs [27], the subscales of the AAA Scale are not independent, and the strongest correlation was found between midwives’ perceptions of discomfort associated with asking and their beliefs about trust (*r* = 0.60). This association highlights the important link between midwives’ conception of the significance of their relationship with the women in their care, and the potential for asking about alcohol use to have a negative impact on this relationship, as has been documented previously [21, 22]. The associations found between beliefs about discomfort and beliefs about harmful alcohol use during pregnancy, attitudes to asking about alcohol use, and behavioural intentions, suggest that beliefs about discomfort are an important area for further inquiry. These beliefs are likely to have important implications for the development of targeted professional development interventions, including underlining the need to provide increased support for midwives to implement strategies for screening and brief intervention that mitigate the potential for negative consequences associated with asking about alcohol use.

Consistent with previous research indicating that midwives considered alcohol screening and brief intervention to be an important part of their role [21, 22], the intention to ask about alcohol use in pregnancy was most strongly related to role beliefs and beliefs about the effectiveness of asking. Furthermore, the associations found between role beliefs and the perceived social norms of managers, and between beliefs about the capacity to ask and the perceived expectations of pregnant women, indicate the potential importance of different normative social influences on midwives’ practice. These associations support the construct validity of the belief subscales, and link these AAA Scale constructs to key determinants of behaviour [19]. Professional development interventions targeting these beliefs may be able to contribute to the effective implementation of policy on screening and brief intervention to address alcohol use in pregnancy.

Beliefs about the capacity to ask about alcohol use were also strongly associated with role beliefs, and perceptions about capacity were more positive among midwives who had completed training on brief intervention in the last 2 years, those who reported using a formal screening tool, and those who reported seeing women earlier in their pregnancy. The cross-sectional nature of this study limits the identification of causal relationships; however, these results suggest that training on brief intervention and the use of a formal screening tool may support midwives’ capacity to ask about alcohol use in pregnancy, as has been demonstrated elsewhere [24]. Establishing opportunities for contact with pregnant women to deliver screening and brief intervention early in pregnancy may also improve midwives’ capacity to ask and advise about alcohol use.

Most midwives believed that brief intervention was effective in identifying women who require support to enable behaviour change, consistent with the evidence that these interventions in the primary care setting are effective and low cost [35]. Midwives’ beliefs about the harmful alcohol use during pregnancy were related to beliefs about role, discomfort, effectiveness, trust and capacity, indicating midwives’ beliefs about harmful alcohol use may be an important influence on beliefs about asking. Review of the evidence supporting recommendations about alcohol use in pregnancy may be an important component of professional development for midwives to promote practices to encourage abstinence from alcohol use in pregnancy.

The methods used in scale development, including use of an established theoretical framework, previous study findings and expert review support the content validity of the AAA Scale. However, despite attempting to recruit all midwives working in the participating health organisation and achievement of a response consistent with similar studies [13], this study is limited by its cross-sectional nature, small sample size and inability to perform an independent confirmatory analysis of the factor structure identified in the exploratory analysis. The study of beliefs within a single health organisation which excludes midwives working in metropolitan areas is also a limitation of this study. Information on the total number of midwives eligible to participate in this study, and on the characteristics of non-responders and the midwifery workforce were not available to the study investigators, limiting the identification of potential sources of response bias which may affect the generalizability of findings.

Although our findings are based on a small sample of midwives, given the strong factor loadings and clear factor structure found, this sample is likely to be sufficient to describe the performance of the scales [36, 37]. We found consistent results when the analysis was limited to the initial 90 respondents, and found no evidence to suggest overfitting, which is typically associated with fragmentation and the identification of additional erroneous factors [38]. Nevertheless, this analysis presents initial scale development findings, and further assessment, including confirmatory evaluation of the scale performance and validation in a large sample of midwives is needed. Ideally this evaluation should be conducted across multiple settings and at multiple points in time.

Further studies are required to confirm the underlying factor structure of the AAA Scale, support generalizability to other settings, and establish whether associations between beliefs and behaviour are causal and can contribute to the design of interventions to support midwives to assist pregnant women to abstain from alcohol use during pregnancy. Evaluation of the practical application of the scale in the professional development context is also needed, and research examining the validity and utility of the scale in the professional development context is currently underway to explore the association between scale constructs and midwives clinical practice using both self report and record audit-based indicators. Use of observational or record audit methods in future studies can address the limitation of common method variance in assessment of behavioural outcomes.

Furthermore, there was limited scope for inclusion of a large number of belief items in this exploratory analysis, and future studies may identify additional salient beliefs relevant to understanding midwives’ practices in asking and advising about alcohol use during pregnancy. The importance of specific beliefs may also vary between practitioner groups due to differences in roles and awareness. In addition, the salience of specific beliefs may change over time as the capacity of health professionals and health systems to respond to the challenges of prevention, diagnosis and management of conditions caused by prenatal alcohol exposure develops.

## Conclusion

We have identified six key constructs underlying midwives beliefs about the assessment of alcohol use during pregnancy. The AAA Scale provides a basis for improved clarity and consistency in the conceptualisation and measurement of midwives’ beliefs about assessing alcohol use during pregnancy. Associations found between established predictors of behaviour and beliefs assessed by the AAA Scale suggests that the scale has the potential to enhance our understanding of factors influencing midwives’ ability to deliver interventions to prevent alcohol use during pregnancy and contribute to the design of professional development interventions to strengthen midwives’ ability to ask and advise about alcohol use in pregnancy.

The constructs established in this exploratory study require confirmatory analysis to support their validity and generalizability, and further studies are required to identify whether the beliefs evaluated by the AAA Scale can make a meaningful contribution to the explanation of midwives behaviour and can be targeted by professional development interventions to support midwives in the delivery of screening and brief intervention for alcohol use during pregnancy. An improved understanding of the constructs underlying midwives attitudes to asking and capacity to ask about alcohol use in pregnancy will have important implications for the development of interventions to promote implementation of clinical practice guidelines.

## Additional file

Additional file 1:
**Alcohol and Pregnancy Questionnaire.** File includes a complete version of the study questionnaire. (PDF 215 kb)

## Abbreviations

AAA Scale: Asking About Alcohol Scale
CI: Confidence interval
FASD: Fetal alcohol spectrum disorder
WA: Western Australia
