# Supplementary material for: Development of a scale to evaluate midwives’ beliefs about assessing alcohol use during pregnancy
Source: BMC Pregnancy Childbirth. 2015 Dec 30;15:353. doi: 10.1186/s12884-015-0779-6 (PMC4696289; doi:10.1186/s12884-015-0779-6)
Supplement: Additional file 1: — Alcohol and Pregnancy Questionnaire. File includes a complete version of the study questionnaire. (PDF 215 kb) [file 12884_2015_779_MOESM1_ESM.pdf]

Alcohol and Pregnancy Questionnaire

The section of the questionnaire asks about you.

1. Are you a midwife?
   - ☐$_0$ no → END – Thank you, only midwives are asked to complete the questionnaire
   - ☐$_1$ yes

2. How long have you worked as a midwife? ____ ____ years

3. How long have you been employed by WACHS as a midwife? ____ ____ years

4. What was the year of your initial graduation from midwifery education? ____ ____ ____ ____ year

5. What is your age? ________ years

6. What is your gender? ☐$_1$ female ☐$_2$ male

7. In which health region do you mainly work? *(choose **one**)*
   - ☐$_1$ Kimberley
   - ☐$_2$ Pilbara
   - ☐$_3$ Midwest
   - ☐$_4$ Goldfields
   - ☐$_5$ Wheatbelt
   - ☐$_6$ South West
   - ☐$_7$ Great Southern
   - ☐$_8$ Other (please specify) ____

The next questions relate to alcohol consumption in pregnancy.

8. Is alcohol consumption during pregnancy associated with any of the following effects on the fetus and child? *(choose **all** that apply)*
   - ☐$_1$ neonatal abstinence syndrome
   - ☐$_2$ delayed development
   - ☐$_3$ learning disabilities
   - ☐$_4$ lowered intelligence
   - ☐$_5$ behavioural problems
   - ☐$_6$ preterm birth
   - ☐$_7$ seizures
   - ☐$_8$ structural brain damage
   - ☐$_9$ spontaneous abortion
   - ☐$_{10}$ Attention Deficit Hyperactivity Disorder (ADHD)
   - ☐$_{11}$ long term emotional disorders
   - ☐$_{12}$ disrupted school experience
   - ☐$_{13}$ alcohol and/or other drug dependence
   - ☐$_{14}$ legal problems
   - ☐$_{15}$ not sure
   - ☐$_{16}$ other (please specify) ____

9. Does alcohol consumption during pregnancy <u>cause</u> any of the following conditions? *(choose **all** that apply)*

☐1 Fetal Alcohol Syndrome (FAS)
☐2 Fetal Alcohol Spectrum Disorder (FASD)
☐3 birth defects
☐4 neurological impairment
☐5 Alcohol-Related Neurodevelopmental Disorder (ARND)
☐6 Attention Deficit Hyperactivity Disorder (ADHD)
☐7 Alcohol-Related Birth Defects (ARBD)
☐8 failure to thrive
☐9 autism
☐10 not sure
☐11 other (please specify) ____

10. Which of the following best describes the <u>general</u> advice you would give to pregnant women regarding alcohol consumption during pregnancy? *(choose **all** that apply)*

☐1 don't become intoxicated
☐2 alcohol is harmful in the first trimester
☐3 try to cut-down drinking alcohol
☐4 not drinking alcohol in pregnancy is the safest option
☐5 drinking alcohol occasionally is OK
☐6 no specific recommendations are given
☐7 other (please specify) ____

11. Do you provide clinical care to pregnant women?

☐1 yes
☐2 no - please specify the reason why you do not provide clinical care ______________________

____ → SKIP TO 132

This section of the questionnaire asks about your <u>current</u> midwifery practice.

12. In which area do you <u>mostly</u> work? *(choose **one**)*

☐1 antenatal care
☐2 labour and birth
☐3 postnatal care
☐4 booking clinic
☐5 other (please specify) ____

13. In which model of care do you <u>mostly</u> work? *(choose **one**)*

☐1 collaborative hospital team
☐2 caseload midwifery
☐3 shared care with general practitioners
☐4 general practitioner led care
☐5 team midwifery
☐6 antenatal midwives' clinic
☐7 community midwifery
☐8 other (please specify) ____

14. In a <u>typical</u> week, approximately how many pregnant women do you care for? ____ ____ number

The next section of the questionnaire relates to your <u>personal opinions</u> about your <u>current midwifery practice</u>. Although some of the questions appear similar,` it is important that you complete all of the questions. <u>There are no right or wrong answers</u>.

For the following items, please tick the circle that best reflects your opinion.

Asking every pregnant woman about whether they have consumed alcohol during their pregnancy is …

15.	useful	○	○	○	○	○	○	○	worthless
16.	harmful	○	○	○	○	○	○	○	beneficial
17.	appropriate	○	○	○	○	○	○	○	inappropriate
18.	unimportant	○	○	○	○	○	○	○	important
19.	pleasant (for me)	○	○	○	○	○	○	○	unpleasant (for me)
20.	good practice	○	○	○	○	○	○	○	bad practice

Please indicate your agreement or disagreement with the following statements.

Asking every pregnant woman about whether they have consumed alcohol during pregnancy …

21. Will threaten my relationship with pregnant women	Strongly agree	○	○	○	○	○	○	○	Strongly disagree
22. Improves women's awareness of the importance of not consuming alcohol during pregnancy	Strongly agree	○	○	○	○	○	○	○	Strongly disagree
23. Will lead to some women feeling judged	Strongly agree	○	○	○	○	○	○	○	Strongly disagree
24. Will identify women who need support to stop consuming alcohol during pregnancy	Strongly agree	○	○	○	○	○	○	○	Strongly disagree
25. Will distress or anger pregnant women	Strongly agree	○	○	○	○	○	○	○	Strongly disagree
26. Is necessary for me to provide appropriate advice and support	Strongly agree	○	○	○	○	○	○	○	Strongly disagree
27. Will cause anxiety and guilt among women who have consumed alcohol during pregnancy	Strongly agree	○	○	○	○	○	○	○	Strongly disagree
28. Will enable changes in behaviour and improved health outcomes for the mother and child	Strongly agree	○	○	○	○	○	○	○	Strongly disagree
29. Will uncover complex problems that are difficult for us to address as midwives	Strongly agree	○	○	○	○	○	○	○	Strongly disagree
30. Offers little real benefit for women who are not in high risk groups	Strongly agree	○	○	○	○	○	○	○	Strongly disagree
31. Could make women fear losing custody of their child	Strongly agree	○	○	○	○	○	○	○	Strongly disagree
32. Will make it easier to diagnose or exclude alcohol related problems in the child	Strongly agree	○	○	○	○	○	○	○	Strongly disagree

Please indicate your agreement or disagreement with the following statements.

33. I intend to ask every pregnant woman about whether they have consumed alcohol during pregnancy	Strongly agree	○	○	○	○	○	○	○	Strongly disagree
34. I don't like asking pregnant women to describe their behaviours which may have harmed their child	Strongly agree	○	○	○	○	○	○	○	Strongly disagree
35. Women's approval of my practice is important to me	Strongly agree	○	○	○	○	○	○	○	Strongly disagree
36. My manager(s) would expect me to ask all pregnant women about whether they have consumed alcohol during pregnancy	Strongly agree	○	○	○	○	○	○	○	Strongly disagree
37. Asking pregnant women about whether they have consumed alcohol during pregnancy can make them feel uncomfortable	Strongly agree	○	○	○	○	○	○	○	Strongly disagree
38. Asking every pregnant woman about whether they have consumed alcohol during pregnancy is up to me	Strongly agree	○	○	○	○	○	○	○	Strongly disagree
39. Pregnant women don't like to be asked about whether they have consumed alcohol during pregnancy	Strongly agree	○	○	○	○	○	○	○	Strongly disagree
40. I have doubts about whether I am doing the right thing when pregnant women who have only consumed small amounts of alcohol experience guilt after discussing their alcohol consumption	Strongly agree	○	○	○	○	○	○	○	Strongly disagree
41. I want to ask every pregnant woman about whether they have consumed alcohol during pregnancy	Strongly agree	○	○	○	○	○	○	○	Strongly disagree
42. Asking pregnant women whether they have consumed alcohol during pregnancy could appear judgemental	Strongly agree	○	○	○	○	○	○	○	Strongly disagree
43. Most pregnant women would expect me to ask every pregnant woman whether they have consumed alcohol during pregnancy	Strongly agree	○	○	○	○	○	○	○	Strongly disagree
44. I don't have sufficient time to ask every pregnant woman about whether they have consumed alcohol during pregnancy	Strongly agree	○	○	○	○	○	○	○	Strongly disagree
45. I feel comfortable asking every pregnant woman whether they have consumed alcohol during pregnancy	Strongly agree	○	○	○	○	○	○	○	Strongly disagree
46. I have the required skills to ask every pregnant woman about whether they have consumed alcohol during pregnancy	Strongly agree	○	○	○	○	○	○	○	Strongly disagree
47. Brief intervention (assessment, feedback, counselling and referral to a specialist if necessary) to decrease alcohol exposure in pregnancy is effective	Strongly agree	○	○	○	○	○	○	○	Strongly disagree
48. Most people whose views I value would approve of me asking all pregnant women whether they have consumed alcohol during pregnancy	Strongly agree	○	○	○	○	○	○	○	Strongly disagree

Please indicate your agreement or disagreement with the following statements.

49. I know exactly what I need to do to ask pregnant women about whether they have consumed alcohol during pregnancy	Strongly agree	○	○	○	○	○	○	○	Strongly disagree
50. Identifying all women who drink any amount of alcohol during pregnancy is good practice	Strongly agree	○	○	○	○	○	○	○	Strongly disagree
51. Asking every pregnant woman about whether they have consumed alcohol during pregnancy is beyond my control	Strongly agree	○	○	○	○	○	○	○	Strongly disagree
52. Most midwives would ask every pregnant woman about whether they have consumed alcohol during pregnancy	Strongly agree	○	○	○	○	○	○	○	Strongly disagree
53. When I discuss the effects on the fetus and child of alcohol consumption during pregnancy I know what to say	Strongly agree	○	○	○	○	○	○	○	Strongly disagree
54. Asking every pregnant woman about whether they have consumed alcohol during pregnancy would be easy	Strongly agree	○	○	○	○	○	○	○	Strongly disagree
55. I have a good understanding of the evidence for the recommendation of no alcohol in pregnancy	Strongly agree	○	○	○	○	○	○	○	Strongly disagree
56. I have sufficient referral resources to adequately address alcohol use problems once identified	Strongly agree	○	○	○	○	○	○	○	Strongly disagree
57. Documentation of any amount of alcohol exposure in pregnancy is important	Strongly agree	○	○	○	○	○	○	○	Strongly disagree
58. Most people who are important to me think that I should ask all pregnant women whether they have consumed alcohol during pregnancy	Strongly agree	○	○	○	○	○	○	○	Strongly disagree
59. I plan to ask every pregnant woman about whether they have consumed alcohol during pregnancy	Strongly agree	○	○	○	○	○	○	○	Strongly disagree
60. Most pregnant women know not to drink alcohol during pregnancy	Strongly agree	○	○	○	○	○	○	○	Strongly disagree
61. I am uncomfortable asking pregnant women about their alcohol consumption when it is the first time I have met them	Strongly agree	○	○	○	○	○	○	○	Strongly disagree
62. I always know what to say when I ask pregnant women about whether they have consumed alcohol during pregnancy	Strongly agree	○	○	○	○	○	○	○	Strongly disagree
63. Women who have consumed alcohol during pregnancy will consider terminating their pregnancy if asked about their alcohol consumption	Strongly agree	○	○	○	○	○	○	○	Strongly disagree
64. Most pregnant women already have good knowledge about alcohol consumption in pregnancy	Strongly agree	○	○	○	○	○	○	○	Strongly disagree
65. When I feel short of time I am less likely to ask pregnant women about whether they have consumed alcohol during pregnancy	Strongly agree	○	○	○	○	○	○	○	Strongly disagree

Please indicate your agreement or disagreement with the following statements.

66. I want my clinical management of pregnant women to be consistent with best practice	Strongly agree	○	○	○	○	○	○	○	Strongly disagree
67. What other midwives think I should do matters to me	Strongly agree	○	○	○	○	○	○	○	Strongly disagree
68. It is difficult to ask pregnant women about alcohol consumption during pregnancy without good knowledge of the effects of alcohol consumption during pregnancy on the fetus and child	Strongly agree	○	○	○	○	○	○	○	Strongly disagree
69. Identifying all women who drink alcohol during pregnancy can help to improve health outcomes	Strongly agree	○	○	○	○	○	○	○	Strongly disagree
70. Most midwives would expect me to ask all pregnant women about whether they have consumed alcohol during pregnancy	Strongly agree	○	○	○	○	○	○	○	Strongly disagree
71. Factors outside my control prevent me from asking every pregnant woman about whether they have consumed alcohol during pregnancy	Strongly agree	○	○	○	○	○	○	○	Strongly disagree
72. I am uncomfortable asking pregnant women about their alcohol consumption before establishing a relationship of trust with them	Strongly agree	○	○	○	○	○	○	○	Strongly disagree
73. It is expected of me that I ask all pregnant women whether they consumed alcohol during pregnancy	Strongly agree	○	○	○	○	○	○	○	Strongly disagree
74. I am confident that I can ask every pregnant woman about whether they consumed alcohol during pregnancy	Strongly agree	○	○	○	○	○	○	○	Strongly disagree
75. I am uncomfortable asking pregnant women about their alcohol consumption as I may have a responsibility to notify the Department of Child Protection	Strongly agree	○	○	○	○	○	○	○	Strongly disagree
76. Pregnant women should completely abstain from consuming alcohol	Strongly agree	○	○	○	○	○	○	○	Strongly disagree
77. Women planning to become pregnant in the near future should abstain from consuming alcohol	Strongly agree	○	○	○	○	○	○	○	Strongly disagree
78. Infrequent consumption of one standard drink of alcohol during pregnancy is not harmful to the mother or fetus	Strongly agree	○	○	○	○	○	○	○	Strongly disagree
79. Information about the effect alcohol may have on the fetus should be readily available to women of childbearing age	Strongly agree	○	○	○	○	○	○	○	Strongly disagree
80. Pregnant women expect midwives to advise them about the consequences of alcohol consumption in pregnancy	Strongly agree	○	○	○	○	○	○	○	Strongly disagree

The next questions are about your <u>current</u> midwifery practice.
Please tick the box or circle that best reflects your practice.

81. Do you ask pregnant women about their alcohol consumption?
    ☐$_0$ no - please specify the reason why you do not ask pregnant women about their alcohol consumption
    ____→ SKIP TO 103
    ☐$_1$ yes

When you ask pregnant women about their alcohol consumption, do you ask about their history of drinking …

82. During their current pregnancy	Always	○	○	○	○	○	○	○	Never
83. For the period before their current pregnancy	Always	○	○	○	○	○	○	○	Never
84. During the period between conception and recognition of their current pregnancy	Always	○	○	○	○	○	○	○	Never

85. Do you ever see pregnant women at their first antenatal visit?
    ☐$_0$ no →SKIP TO 88
    ☐$_1$ yes

86. In a <u>typical</u> week, how many pregnant women do you see for their first antenatal visit?

    ____ ____ number

87. At the first antenatal visit, do you ask pregnant women about their alcohol consumption in pregnancy?	Always	○	○	○	○	○	○	○	Never

88. Do you ever see pregnant women at their booking visit?
    ☐$_0$ no →SKIP TO 90
    ☐$_1$ yes

89. At the booking visit, do you ask pregnant women about their alcohol consumption in pregnancy?	Always	○	○	○	○	○	○	○	Never

90. Do you ever see pregnant women during their first trimester of pregnancy?
    ☐$_0$ no →SKIP TO 92
    ☐$_1$ yes

91. When you see pregnant women during the first trimester of pregnancy, do you ask them about their alcohol consumption in pregnancy?	Always	○	○	○	○	○	○	○	Never

92. Do you ever see pregnant women during their second trimester of pregnancy?
    ☐$_0$ no →SKIP TO 94
    ☐$_1$ yes

93. When you see pregnant women during the second trimester of pregnancy, do you ask them about their alcohol consumption in pregnancy?	Always	○	○	○	○	○	○	○	Never

94. Do you ever see pregnant women during their third trimester of pregnancy?
☐$_0$ no →SKIP TO 96
☐$_1$ yes

95. When you see pregnant women during the third trimester of pregnancy, do you ask them about their alcohol consumption in pregnancy? Always ○ ○ ○ ○ ○ ○ ○ Never

Do you use any of the following screening tools to assess alcohol consumption during pregnancy?

96.	WACHS Alcohol AUDIT Tool (Form MR202E)	☐$_1$ Yes	☐$_0$ No
97.	AUDIT-C (Alcohol Use Disorders Identification Test - Consumption)	☐$_1$ Yes	☐$_0$ No
98.	T-ACE (Tolerance, Annoyed, Cut down, Eye-opener)	☐$_1$ Yes	☐$_0$ No
99.	TWEAK (Tolerance, Worry, Eye-opener, Amnesia, Cut-down)	☐$_1$ Yes	☐$_0$ No
100.	CAGE (Cut down, Annoyed, Guilty, Eye-opener)	☐$_1$ Yes	☐$_0$ No
101.	MAST (Michigan Alcoholism Screening Test)	☐$_1$ Yes	☐$_0$ No

102. other (please specify) ____

103. Do you provide information to pregnant women about the effects on the fetus and child of consuming alcohol during pregnancy? Always ○ ○ ○ ○ ○ ○ ○ Never

104. Are you aware of policies, procedures or operational directives in WACHS advising assessment of alcohol consumption during pregnancy? ☐$_1$ yes ☐$_0$ no

105. If indicated, do you conduct brief intervention for alcohol consumption in pregnancy? ☐$_1$ yes ☐$_0$ no

106. Did you receive brief intervention training for alcohol consumption during pregnancy at your orientation? ☐$_1$ yes ☐$_0$ no

107. Have you completed brief intervention training for alcohol consumption during pregnancy as part of professional development within the last 2 years? ☐$_1$ yes ☐$_0$ no

Are you <u>confident</u> that you can …

108. Assess alcohol consumption during pregnancy	Very confident	○	○	○	○	○	○	○	Not at all confident
109. Advise pregnant women about alcohol consumption during pregnancy	Very confident	○	○	○	○	○	○	○	Not at all confident
110. Explain to pregnant women the effects on the fetus and child of alcohol consumption during pregnancy	Very confident	○	○	○	○	○	○	○	Not at all confident
111. Conduct brief intervention for alcohol consumption during pregnancy	Very confident	○	○	○	○	○	○	○	Not at all confident

Do you have sufficient <u>time</u> to …

112. Assess alcohol consumption during pregnancy	Always	○	○	○	○	○	○	○	Never
113. Advise pregnant women about alcohol consumption during pregnancy	Always	○	○	○	○	○	○	○	Never
114. Explain to pregnant women the effects on the fetus and child of alcohol consumption during pregnancy	Always	○	○	○	○	○	○	○	Never
115. Conduct brief intervention for alcohol consumption during pregnancy	Always	○	○	○	○	○	○	○	Never

Could you offer greater <u>support</u> to pregnant women if you had …

116. More involvement in antenatal care	Always	○	○	○	○	○	○	○	Never
117. More involvement in early pregnancy care	Always	○	○	○	○	○	○	○	Never
118. Standardised forms to record health, medical and alcohol consumption history	Always	○	○	○	○	○	○	○	Never
119. More knowledge about conversational techniques to use when assessing alcohol consumption in pregnancy	Always	○	○	○	○	○	○	○	Never
120. Prompts for brief intervention	Always	○	○	○	○	○	○	○	Never
121. More accessible specialist alcohol services for referring pregnant women to	Always	○	○	○	○	○	○	○	Never
122. Greater supply of written materials to give to pregnant women	Always	○	○	○	○	○	○	○	Never
123. More standardised resources to use when running antenatal classes or parenting education programs	Always	○	○	○	○	○	○	○	Never
124. Web-based educational resources for midwives about alcohol consumption during pregnancy and fetal alcohol spectrum disorder	Always	○	○	○	○	○	○	○	Never
125. Hard-copy educational resources for midwives about alcohol consumption during pregnancy and fetal alcohol spectrum disorder	Always	○	○	○	○	○	○	○	Never
126. Professional development about alcohol consumption during pregnancy and fetal alcohol spectrum disorder	Always	○	○	○	○	○	○	○	Never
127. Professional development about screening tools for alcohol consumption	Always	○	○	○	○	○	○	○	Never
128. Professional development about brief intervention	Always	○	○	○	○	○	○	○	Never

129. Please list specialised alcohol services that you can access for referring pregnant women.

____

____

____

____

130. Please list resources that you can access to give to pregnant women on alcohol consumption during pregnancy and fetal alcohol spectrum disorder.

____

____

____

____

131. Are there any other comments that you would like to make?

____

____

____

____

132. Have you started filling in the online version of this questionnaire but not completed it? ☐1 Yes ☐0 No

IF YES, would you please give us your email address so we don't count your responses twice?

____ email address

133. Do you wish to be included in the prize draw for the weekend away for two? ☐1 Yes ☐0 No

IF YES, please record your HE number _______________ HE number

Thank you for taking the time to participate in this study and complete the questionnaire. The information you provided will be used to support strategies to prevent fetal alcohol spectrum disorder, and we thank you for your valuable contribution to this process.

If you would like more information on alcohol and pregnancy and fetal alcohol spectrum disorder please visit our website http://alcoholpregnancy.childhealthresearch.org.au/ or contact Jan Payne on 9489 7752 or midwives@ichr.uwa.edu.au.
